# Influenza Vaccination Status and Clinical Outcomes Among Adults Hospitalized with Community-Acquired Pneumonia: A Retrospective Cohort from Saudi Arabia

**DOI:** 10.3390/vaccines14090810

**Published:** 2026-09-14

**Authors:** Shouq M. Alzaaqi, Ayman Banjar, Majed A. Almoghrabi, Seham F. Basheer, Sami Alsaedi, Saeed M. Algarni, Ezzuddin A. Okmi, Sarah A. Barzanji, Samah A. Bukhari, Abeer S. Alasmari, Abdullah M. Alyemeny, Abdulaziz A. Alshaalan, Haytham A. Sheerah

**Affiliations:** 1 King Abdullah International Medical Research Center, King Saud bin Abdulaziz University for Health Sciences, Ministry of National Guard Health Affairs, Riyadh 11481, Saudi Arabia; 2 Global Health Directorate, Public Health Authority, Riyadh 13351, Saudi Arabia; 3 Population Health Deputyship, Ministry of Health, Riyadh 11176, Saudi Arabia; 4 Department of Otorhinolaryngology, Imam Abdulrahman Alfaisal Hospital, Ministry of Health, Riyadh 14723, Saudi Arabia; 5 Communicable Diseases Sector, Public Health Authority, Riyadh 13351, Saudi Arabia; 6 Department of Endodontics, King Abdulaziz Medical City, Ministry of National Guard Health Affairs, Jeddah 9515, Saudi Arabia; 7 Department of Periodontics, King Abdulaziz Medical City, Ministry of National Guard Health Affairs, Jeddah 9515, Saudi Arabia; 8 Department of Respiratory Services, King Abdulaziz Medical City, Ministry of National Guard Health Affairs, Riyadh 11426, Saudi Arabia; 9 International Collaboration Department at the Chief Executive Office, Public Health Authority, Riyadh 13351, Saudi Arabia

**Keywords:** influenza vaccination, community-acquired pneumonia, influenza, intensive care unit, mortality, length of stay

## Abstract

Background: Influenza vaccination is widely recommended to prevent influenza and its complications; however, its association with clinical outcomes among high-risk populations remains uncertain. This study aimed to investigate the association between influenza vaccination status and clinical outcomes among adults hospitalized with community-acquired pneumonia (CAP) in Saudi Arabia. Methods: This retrospective cohort study included adults hospitalized with CAP in Saudi public hospitals between October 2023 and March 2024. Data on demographic characteristics, comorbidities, influenza vaccination status, laboratory-confirmed influenza infection, intensive care unit (ICU) admission, in-hospital mortality, and length of hospital stay were obtained from electronic health records. Logistic regression analyses were performed to evaluate the associations between influenza vaccination and clinical outcomes. Results: Among 485 patients, 114 (23.5%) had received the seasonal influenza vaccine. The prevalence of laboratory-confirmed influenza infection was similar among vaccinated and unvaccinated patients (57.9% vs. 58.5%, *p*-value = 0.910). Vaccinated patients were less likely to require ICU admission (1.8% vs. 7.5%, *p*-value = 0.025), while no significant differences were observed in length of hospital stay or in-hospital mortality. After adjustment for age, sex, and comorbidities, influenza vaccination was associated with lower odds of ICU admission (OR = 0.200, 95% CI: 0.045, 0.894). No significant associations were observed between influenza vaccination and laboratory-confirmed influenza infection, length of hospital stay, or in-hospital mortality. Conclusions: Among adults hospitalized with CAP in Saudi Arabia, influenza vaccination was associated with a lower likelihood of ICU admission, supporting its potential role in reducing disease severity among high-risk populations.

## 1. Introduction

Community-acquired pneumonia (CAP) is a major cause of hospitalization, intensive care unit (ICU) admission, and death worldwide, with the greatest burden occurring among older adults and people with underlying chronic conditions [1,2]. Influenza viruses contribute substantially to the burden of CAP through both primary viral pneumonia and secondary bacterial infections following influenza illness [3]. Seasonal influenza epidemics are associated with increased healthcare utilization, excess hospital admissions, and elevated mortality rates, placing considerable pressure on healthcare systems [4].

Annual influenza vaccination is recommended for individuals at increased risk of influenza-related complications and has been shown to reduce laboratory-confirmed influenza infection and influenza-associated hospitalization [5,6,7,8]. A meta-analysis of 30 studies reported a 41% reduction in laboratory-confirmed influenza-associated hospitalization among vaccinated adults [9]. Vaccination has also been associated with more favorable outcomes among hospitalized patients, including lower mortality, reduced pneumonia severity, decreased ICU utilization, and shorter hospital stays [8,10,11,12,13,14,15,16,17,18]. In a meta-analysis of 15 studies involving community-dwelling older adults, influenza vaccination was associated with a 33% reduction in hospitalization for pneumonia, a 47% reduction in mortality following hospitalization for pneumonia, and a 50% reduction in all-cause mortality [19]. However, other studies have reported no significant associations with hospitalization outcomes, disease severity, or mortality [20,21,22,23]. These inconsistent findings may reflect differences in study populations, healthcare systems, circulating influenza strains, and vaccine effectiveness across seasons, highlighting the need for additional evidence from different geographical and healthcare settings.

This issue is particularly relevant in Saudi Arabia, where seasonal influenza circulates annually and millions of international pilgrims visit the country during Hajj and Umrah, creating conditions that facilitate the transmission of respiratory pathogens [24,25]. As a result, influenza prevention represents an important public health priority, and annual influenza vaccination is strongly recommended for individuals at increased risk of complications [24,26]. Despite the public health importance of influenza prevention and ongoing vaccination campaigns, evidence regarding the relationship between influenza vaccination and clinical outcomes among hospitalized patients is lacking in Saudi Arabia and the Gulf area. Most available studies have been conducted in Europe and North America, and their findings may not be directly generalizable to Saudi Arabia or the broader Gulf area because of differences in population characteristics, healthcare utilization patterns, vaccination uptake, and epidemiological factors [27,28]. Importantly, local evidence is particularly limited regarding whether prior influenza vaccination is associated with the severity and clinical course of CAP among patients who nevertheless require hospitalization. In this context, this study aimed to examine whether influenza vaccination status was associated with clinical outcomes among adults hospitalized with CAP in Saudi Arabia, including laboratory-confirmed influenza infection, ICU admission, in-hospital mortality, and length of hospital stay.

## 2. Methods

### 2.1. Study Design and Participants

This hospital-based retrospective cohort study analyzed data from adult patients admitted with CAP to Saudi public hospitals between October 2023 and March 2024. Information was obtained from routinely collected electronic health records on 5 December 2024. Eligible participants were patients aged ≥18 years who required hospitalization due to CAP during the study period. CAP was identified based on the diagnosis documented in the electronic health records at the time of hospital admission. Because data were retrospectively obtained from multiple hospitals, the specific clinical and radiological criteria used to establish the diagnosis were not available for individual patients. Patients with incomplete information on influenza vaccination status, ICU admission, mortality, or length of hospital stay were excluded. Of the 534 available records, 485 patients met the eligibility criteria and were included in the final analysis.

### 2.2. Data Collection and Study Variables

Demographic and clinical information was retrieved from hospital records. Demographic characteristics included age and sex. Clinical variables included a history of diabetes mellitus, hypertension, chronic kidney disease, asthma, and ischemic heart disease. Information on prior receipt of seasonal influenza vaccination was retrieved from the same records. Study outcomes included laboratory-confirmed influenza infection, ICU admission during hospitalization, in-hospital mortality, and length of hospital stay. Influenza infection was confirmed by polymerase chain reaction (PCR) testing as reported in the electronic health records. Information on the specific PCR assay, respiratory specimen type, and testing criteria was not consistently available across participating hospitals. Length of stay was calculated as the number of days between hospital admission and discharge.

The specific vaccine product, manufacturer, and antigenic composition administered to individual patients were not available in the electronic health records. Therefore, vaccination exposure was based only on documented receipt of seasonal influenza vaccination before the index hospitalization. However, for contextual purposes, the WHO-recommended influenza vaccine compositions for the relevant Northern Hemisphere seasons were mentioned elsewhere [29,30,31].

### 2.3. Statistical Analysis

Patient characteristics were summarized according to influenza vaccination status. Continuous variables were reported as mean ± standard deviation (SD), whereas categorical variables were presented as frequencies and percentages. Comparisons between vaccinated and unvaccinated patients were performed using the independent-samples *t*-test for continuous variables and the chi-square test or Fisher’s exact test for categorical variables. Logistic regression analyses were conducted to evaluate the association between influenza vaccination and the study outcomes. Odds ratios (ORs) and 95% confidence intervals (CIs) were estimated. Three models were examined: Model I included no covariate adjustment; Model II adjusted for age and sex; and Model III additionally adjusted for comorbidities that showed significant differences in univariate analyses. Statistical analyses were performed using IBM SPSS Statistics version 22.0 (IBM Corp., Armonk, NY, USA). Statistical significance was defined as a two-tailed *p*-value < 0.05.

## 3. Results

### 3.1. Participant Characteristics

A total of 485 patients hospitalized with CAP were included in the analysis, of whom 114 (23.5%) had received the seasonal influenza vaccine and 371 (76.5%) had not. The mean age of participants was 52.1 ± 22.4 years, and 45.2% were men. No significant differences were observed regarding age, sex, diabetes, asthma, or ischemic heart disease between vaccinated and unvaccinated patients (*p*-values > 0.05). However, vaccinated patients had a higher prevalence of hypertension (15.8% vs. 5.4%, *p*-value < 0.001) and chronic kidney disease (8.8% vs. 1.9%, *p*-value < 0.001) compared with unvaccinated patients (Table 1).

### 3.2. Clinical Outcomes According to Influenza Vaccination Status

The proportion of patients with laboratory-confirmed influenza infection was similar between vaccinated and unvaccinated patients (57.9% vs. 58.5%, *p*-value = 0.910). The proportion of patients hospitalized for >4 days was comparable between vaccinated and unvaccinated patients (28.1% vs. 32.3%, *p*-value = 0.389). Overall, ICU admission was recorded in 6.2% of patients and was significantly less frequent among vaccinated patients than among unvaccinated patients (1.8% vs. 7.5%, *p*-value = 0.025). In-hospital mortality was recorded in 7.6% of patients and was lower among vaccinated patients (4.4% vs. 8.6%), although this difference did not reach statistical significance (*p*-value = 0.136) (Table 2).

### 3.3. Association Between Influenza Vaccination and Clinical Outcomes

Influenza vaccination was associated with lower odds of ICU admission in the unadjusted analysis (OR = 0.219, 95% CI: 0.051, 0.933). This association remained significant after adjustment for age and sex (OR = 0.213, 95% CI: 0.049, 0.928) and after further adjustment for hypertension and chronic kidney disease (OR = 0.200, 95% CI: 0.045, 0.894). However, influenza vaccination was not significantly associated with laboratory-confirmed influenza infection in either the crude analysis (OR = 0.976, 95% CI: 0.638, 1.493) or after adjustment for age and sex (OR = 0.887, 95% CI: 0.557, 1.412) or further adjustment for hypertension and chronic kidney disease (OR = 0.873, 95% CI: 0.542, 1.406). Similarly, influenza vaccination was not significantly associated with prolonged hospitalization (>4 days). The crude OR was 0.816 (95% CI: 0.514, 1.297), which remained non-significant after adjustment for age and sex (OR = 0.856, 95% CI: 0.522, 1.405) and after further adjustment for hypertension and chronic kidney disease (OR = 0.763, 95% CI: 0.455, 1.279). Similarly, no significant association was observed between influenza vaccination and in-hospital mortality. The crude OR for mortality was 0.486 (95% CI: 0.185, 1.278), while the corresponding adjusted ORs were 0.484 (95% CI: 0.179, 1.309) and 0.490 (95% CI: 0.178, 1.348) (Table 3).

## 4. Discussion

In this retrospective cohort study of adults hospitalized with CAP in Saudi Arabia, influenza vaccination was associated with a substantially lower likelihood of ICU admission, even after adjustment for age, sex, and comorbidities.

Several mechanisms may explain the observed association between influenza vaccination and lower ICU admission. Although influenza vaccination is primarily intended to prevent influenza infection, previous studies have suggested that vaccination may also attenuate disease severity among individuals who develop respiratory infections despite vaccination. Vaccinated patients may experience less severe pulmonary inflammation, lower risk of respiratory complications, and reduced susceptibility to secondary bacterial infections than unvaccinated patients [3,8,10,13]. These mechanisms could reduce the likelihood of clinical deterioration requiring intensive care.

Our findings are broadly consistent with previous studies reporting reduced disease severity among vaccinated individuals. In a multicenter study of 2368 patients with CAP, Tessmer et al. reported that influenza vaccination was associated with reduced pneumonia severity, as measured by the Confusion, Urea, Respiratory Rate, and Blood Pressure (CURB) score (OR = 0.76, 95% CI: 0.60, 0.98) [12]. Thompson et al. reported lower risks of influenza-associated ICU admission among vaccinated adults (OR = 0.41, 95% CI: 0.18, 0.96) in 3135 hospitalized patients with acute respiratory illness [13]. Similarly, Loubet et al. reported a lower likelihood of ICU admission among vaccinated patients hospitalized with influenza (OR = 0.50, 95% CI: 0.28, 0.90) [16]. In addition, Mena et al. found that influenza vaccination was associated with a lower risk of serious outcomes, including ICU admission and/or mortality, among 551 adults hospitalized with influenza (OR = 0.27, 95% CI: 0.11, 0.65) [14].

In contrast, we did not observe significant associations between influenza vaccination and laboratory-confirmed influenza infection, length of stay, or in-hospital mortality. Similar null findings have been reported by several previous studies. Joshi et al. found no significant association between influenza vaccination and influenza-related respiratory failure or mortality among hospitalized older adults [21], while Heidemann et al. observed largely similar hospitalization outcomes among vaccinated and unvaccinated adults hospitalized with influenza A [23]. McLean et al. also reported no significant association between influenza vaccination and hospitalization among adults with laboratory-confirmed influenza infection [22]. However, our findings differ from those of previous studies that reported lower mortality, shorter hospital stays, or both among vaccinated patients hospitalized with influenza or pneumonia [9,10,15,17].

The lack of association between influenza vaccination and laboratory-confirmed influenza infection in our study should be interpreted cautiously because the study included only hospitalized patients with CAP rather than the general population. Likewise, although in-hospital mortality was numerically lower among vaccinated than unvaccinated patients (4.4% vs. 8.6%), this difference did not reach statistical significance (*p*-value = 0.136). The relatively small number of deaths and vaccinated patients may have limited statistical power, and a larger study would provide greater power to determine whether this observed difference represents a true association. It is also possible that influenza vaccination exerts a greater influence on disease severity, as reflected by ICU admission, than on later outcomes such as survival.

Of note, our results have important clinical and public health implications. ICU admission is a major indicator of disease severity and is associated with increased healthcare costs, resource utilization, and risk of adverse outcomes [32]. Therefore, reducing the need for intensive care may lessen the burden on healthcare systems while improving patient outcomes. Although influenza vaccination is primarily recommended to prevent influenza infection and its complications [5,6], our findings suggest that prior vaccination may also be associated with lower disease severity among hospitalized patients with CAP. These findings provide region-specific evidence from Saudi Arabia, where data on influenza vaccination and clinical outcomes among hospitalized patients with CAP remain limited. This observation is particularly relevant in Saudi Arabia, where seasonal influenza circulates annually and large numbers of international pilgrims visit the country each year [24].

Several limitations should be considered when interpreting our findings. First, the retrospective design precludes establishing causal relationships between influenza vaccination and clinical outcomes. Second, the study relied on routinely collected electronic health records, which may be subject to incomplete documentation, coding inaccuracies, or residual measurement error. Third, information on several potentially important confounders was unavailable, including smoking status, socioeconomic characteristics, severity of pneumonia at presentation, time since vaccination, and vaccine type. Fourth, the relatively small number of vaccinated patients and clinical events, particularly ICU admissions and deaths, may have limited statistical power to detect modest associations with some outcomes. Consequently, non-significant findings for mortality should be interpreted cautiously. The limited number of events also restricted our ability to perform adequately powered subgroup analyses, including analyses according to laboratory-confirmed influenza status and other patient characteristics. Fifth, the study included only patients hospitalized in Saudi public hospitals, which may limit the generalizability of the findings to private healthcare settings or other countries. Sixth, because only hospitalized patients were included, the study could not evaluate the effectiveness of influenza vaccination in preventing pneumonia-related hospitalization. Seventh, the limited number of vaccinated patients and clinical events precluded adequately powered subgroup analyses according to laboratory-confirmed influenza status. Eighth, the exact interval between influenza vaccination and hospital admission was not available in the extracted records; therefore, it was not possible to restrict the vaccinated group to patients who had received the vaccine at least 14 days before admission.

## 5. Conclusions

Among adults hospitalized with CAP in Saudi Arabia, influenza vaccination was associated with a significantly lower likelihood of ICU admission. However, influenza vaccination was not significantly associated with laboratory-confirmed influenza infection, length of hospital stay, or in-hospital mortality. Efforts to improve influenza vaccination uptake, particularly among high-risk populations, should remain a public health priority. Future prospective multicenter studies with larger sample sizes and more detailed clinical information are needed to confirm these findings.

## Figures and Tables

**Table 1:** Sociodemographic characteristics and medical history of participants by their influenza vaccination status.

Characteristics		Overall	Unvaccinated	Vaccinated	*p*-Value
Number of patients		485	371	114	--
Sex, %	Men	219 (45.2)	166 (44.7)	53 (46.5)	0.743
	Women	266 (54.8)	205 (55.3)	61 (53.5)	
Age, years (mean ± SD)		52.1 ± 22.4	52.6 ± 22.3	50.6 ± 22.8	0.395
Age, %	≤60 years	293 (60.4)	218 (58.8)	75 (65.8)	0.180
	>60 years	192 (39.6)	153 (41.2)	39 (34.2)	
Hypertension, %		38 (7.8)	20 (5.4)	18 (15.8)	<0.001
Diabetes, %		30 (6.2)	20 (5.4)	10 (8.8)	0.190
Asthma, %		23 (4.7)	16 (4.3)	7 (6.1)	0.422
Chronic kidney disease, %		17 (3.5)	7 (1.9)	10 (8.8)	<0.001
Ischemic heart disease, %		12 (2.5)	8 (2.2)	4 (3.5)	0.416

**Table 2:** Clinical outcomes of participants by their influenza vaccination status.

Characteristics		Overall	Unvaccinated	Vaccinated	*p*-Value
Number of patients		485	371	114	--
Laboratory-confirmed influenza		283 (58.4)	217 (58.5)	66 (57.9)	0.910
Length of hospital stay, %	≤4 days	333 (68.7)	251 (67.7)	82 (71.9)	0.389
	>4 days	152 (31.3)	120 (32.3)	32 (28.1)	
ICU admission, %		30 (6.2)	28 (7.5)	2 (1.8)	0.025
Mortality, %		37 (7.6)	32 (8.6)	5 (4.4)	0.136

**Table 3:** The association between influenza vaccination and clinical outcomes.

Clinical Outcomes	Model I OR (95% CI)	Model II OR (95% CI)	Model III OR (95% CI)
Laboratory-confirmed influenza	0.976 (0.638, 1.493)	0.887 (0.557, 1.412)	0.873 (0.542, 1.406)
Length of hospital stay > 4 days	0.816 (0.514, 1.297)	0.856 (0.522, 1.405)	0.763 (0.455, 1.279)
ICU admission	0.219 (0.051, 0.933)	0.213 (0.049, 0.928)	0.200 (0.045, 0.894)
Mortality	0.486 (0.185, 1.278)	0.484 (0.179, 1.309)	0.490 (0.178, 1.348)

Model I: Unadjusted; Model II: Adjusted for age and sex; and Model III: Further adjusted for hypertension and chronic kidney disease.

## Data Availability

Upon reasonable request, data will be provided by the corresponding author.
